# Applying Machine Learning to Construct a Model of Risk of Depression in Patients Following Cardiac Surgery with the Use of the SF-12 Survey

**DOI:** 10.3390/ijerph20064876

**Published:** 2023-03-10

**Authors:** Katarzyna Nowicka-Sauer, Krzysztof Jarmoszewicz, Andrzej Molisz, Krzysztof Sobczak, Marta Sauer, Mariusz Topolski

**Affiliations:** 1 Department of Family Medicine, Faculty of Medicine, Medical University of Gdańsk, Dębinki 2 Str., 80-211 Gdańsk, Poland; 2 Department of Cardiac Surgery, Kashubian Centre for Cardiac and Vascular Diseases, Ceynowa Specialist Hospital, Jagalskiego 10 Str., 84-200 Wejherowo, Poland; jarmo38@gmail.com; 3 Department of Otolaryngology, University Clinical Centre, Medical University of Gdańsk, Smoluchowskiego 17 Str., 80-214 Gdansk, Poland; amol@gumed.edu.pl; 4 Division of Medical Sociology and Social Pathology, Faculty of Health Sciences, Medical University of Gdańsk, Tuwima 15 Str., 80-210 Gdańsk, Poland; krzysztof.sobczak@gumed.edu.pl; 5 Radiation Protection Office, University Clinical Centre, Medical University of Gdańsk, Smoluchowskiego 17 Str., 80-214 Gdańsk, Poland; msauer@uck.gda.pl; 6 Department of Systems and Computer Networks, Faculty of Information and Communication Technology, Wroclaw University of Science and Technology, Janiszewskiego 11/17 Str., 50-372 Wroclaw, Poland; mariusz.topolski@pwr.edu.pl

**Keywords:** cardiac surgery, patients reported outcomes (PROs), depression, risk factors, machine learning

## Abstract

Background: Depression is a common problem in patients with cardiovascular diseases. Identifying a risk factor model of depression has been postulated. A model of the risk of depression would provide a better understanding of this disorder in this population. We sought to construct a model of the risk factors of depression in patients following cardiac surgery, with the use of machine learning. Methods and Measures: Two hundred and seventeen patients (65.4% men; mean age 65.14 years) were asked to complete the short form health survey-12 (SF-12v.2), three months after hospital discharge. Those at risk of depression were identified based on the SF-12 mental component summary (MCS). Centroid class principal component analysis (CCPCA) and the classification and regression tree (CART) were used to design a model. Results: A risk of depression was identified in 29.03% of patients. The following variables explained 82.53% of the variance in depression risk: vitality, limitation of activities due to emotional problems (role-emotional, RE), New York Heart Association (NYHA) class, and heart failure. Additionally, CART revealed that decreased vitality increased the risk of depression to 45.44% and an RE score > 68.75 increased it to 63.11%. In the group with an RE score < 68.75, the NYHA class increased the risk to 41.85%, and heart failure further increased it to 44.75%. Conclusion: Assessing fatigue and vitality can help health professionals with identifying patients at risk of depression. In addition, assessing functional status and dimensions of fatigue, as well as the impact of emotional state on daily functioning, can help determine effective intervention options.

## 1. Introduction

Depression is a common problem in outpatients with various somatic conditions [1]. Recent studies have provided further evidence that depression following cardiac surgery remains a serious problem and one that is often overlooked. The prevalence of depression in this particular group of patients varies between studies and ranges from 13% to 40% of patients. In some patients, it may be a long-term problem, becoming a chronic condition [2,3,4]. The increasing number of cardiac surgeries worldwide is leading to a large group of people suffering from depression after cardiac surgery.

Apart from the psychological suffering and decreased function of these patients, depression also increases the risk of rehospitalization, morbidity, and mortality [4,5,6]. Screening for depression is recommended by the American Heart Association (AHA) and the American Psychiatrists Association (APA) [7] both pre- and post-operatively [2,4,8]. Identifying patients at risk of depression is vital for implementing early interventions [3]. Considering the higher probability of depression post-operatively, mental health monitoring following surgery seems to be essential [2,3,4]. Screening is a first crucial step in identifying mental health problems. In addition, understanding predictors and risk factors of depression is the next step in clarifying the underlying mechanisms and planning more effective management.

Although no single specific method has been proven to improve cardiac prognosis in the case of coexisting depression, treatment of depression is highly recommended. Psychotherapy with special emphasis on cognitive-behavioral therapy (CBT) and/or pharmacotherapy is recommended, according to the severity of the depressive symptoms. Depression and anxiety symptoms were more significantly decreased with longer-lasting interventions and when provided following surgery by trained health care providers [9]. Despite a substantial body of studies on depression in those with cardiovascular diseases, further investigations in the field of treatment have been recommended [6]. Psychotherapy is an effective method of treatment, especially in patients with mild to moderate symptoms. The specific feelings, concerns, or symptoms that are the most burdensome for patients must be identified. They should be addressed as early as possible in the process of psychotherapy. Such an analysis is in accordance with a more patient-centered approach, to make our interventions as effective as possible [6]. Here, machine learning can be used for statistics that, with the help of advanced data analysis, enable the creation of predictive models. The advantage of modelling is that the results obtained have stronger discriminant power in the prediction task than those of the classical methods based on multiple logistic regression analysis [10,11]. Many studies have suggested that machine learning is an effective statistical approach in medical sciences, including for depression prediction [12,13,14,15]. To the best of our knowledge, no prior studies have explored depression risk modeling with the use of machine learning in patients following cardiac surgery. Such an in-depth analysis would provide health professionals with a better understanding of a particular disorder and the patient’s perspective. By using this technique, we can identify the most burdensome or alarming symptom(s), which may be omitted or ignored during routine assessment. Thus, it can provide health professionals with additional knowledge about the symptoms for which patients should be screened, to contribute to the early detection of a disorder [16]. As a consequence, this may prevent subsequent unnecessary patient suffering and improve their quality of life.

Our two major aims with the present study were, first, to estimate the risk of depression in patients following cardiac surgery; second, to identify factors that may predict depression risk with the use of machine learning methods.

## 2. Methods

### 2.1. Participants

The study involved 217 patients, who completed a follow-up visit after at least three months following cardiac surgery (Table 1).

### 2.2. Measures

Patients were asked to complete the Medical Outcomes Trust Short Form Health Survey-12 (SF-12v.2). The SF-12 is derived from the SF-36, which is one of the most frequently used methods for the assessment of health-related quality of life [17]. The SF-12 allows assessing several dimensions of patient-reported outcomes (PROs), which are grouped in the following domains: physical functioning (PF), role-physical (role limitation because of physical health problems, RP), bodily pain (BP), general health (GH), vitality (VT), social functioning (SF), role-emotional (limitation of activities due to emotional problems, RE), and mental health (MH). In addition, two meta-scores can be obtained with the use of the SF-12: mental component summary (MCS) and physical component summary (PCS) [18].

Socio-demographic data were gathered with the use of a tailor-made questionnaire. Clinical data were gathered in a review of medical files.

The study report was conducted in accordance with the strengthening the reporting of observational studies in epidemiology (STROBE) guidelines.

### 2.3. Assessment of Risk of Depression Based on SF-12

According to the SF-12 manual, the mental component summary (MCS) score allows the determination of the risk of depression. An MCS score below 42 points indicates a risk of depression [18].

### 2.4. Ethical Approval and Statement of Informed Consent

The study was approved by the Independent Bioethics Commission for Research of Medical University of Gdańsk. Written informed consent was obtained from all the patients. Data are available upon request from the corresponding author.

### 2.5. Statistical Analysis

Quantitative variables are presented as mean values, standard deviations, or absolute values and percentage. Pearson’s product-moment correlation coefficient was calculated for all correlations. A level of significance of α ≤ 0.05 was used. The current study was a study with a targeted sample. The sample size was estimated after the study was performed, as follows: assumed population: 500 patients, with 95% confidence level, fraction size 0.5, with a maximum error of 5%. Principal component analysis (PCA) is an unsupervised linear transformation technique commonly used to reduce dimensionality. A PCA identifies patterns in data based on correlations between features. The task of a PCA is to search for directions of maximum variance in a multidimensional space and to project them into a new subspace containing as many (or fewer) dimensions as the original feature space. The orthogonal axes (principal components) projected into the new subspace symbolize the directions of maximum variance, under the introduced constraint that the new feature axes are perpendicular to each other [19]. Kernel principal component analysis (KPCA) is an algorithm used to solve the problem of nonlinear dimensionality reduction [20]. Linear discriminant analysis (LDA) works similarly to principal component analysis. It performs a projection of the feature space onto a lower dimensional space. Unlike PCA, this algorithm aims to maximize the differences between the classes. This algorithm belongs to the supervised methods. For its correct operation, we must know the classes to which the objects belong, e.g., the level of risk. Two models were used in this study: centroid class principal component analysis (CCPCA) [11,21,22] and gradient component analysis (GPCA) [23]. The centroid class principal component analysis (CCPCA) model is a modification of PCA. The main assumption of the concept is the rotation of a normalized feature space of the objects, according to the classes’ centroids. The main goal of such an approach is the separation of the subspaces of features that are oriented in classes. The class in the problem is the different level of risk. The GPCA is a concept for building the main components in the PCA method; for this purpose, the stochastic gradient optimization method was used [23]. The final model was built using classification and regression trees (CART), which is a modern form of decision trees. CART is used for classification and prediction problems. In the present study, CART was used for risk prediction. These models were obtained by partitioning the data space and fitting a simple prediction model within each partition. This was performed recursively. We used Statistica 13 by StatSoft and Statistical Package for the Social Sciences (SPSS) 27 for the statistical analyses.

## 3. Results

### 3.1. Baseline Characteristics of the Study Population

A total of 217 patients (65.4% of men) with mean age 65.14 years were enrolled in the study, including 56.7% patients following coronary artery by-pass grafting (off-pump or on-pump), 20.3% single not CABG procedure and 23.0% following combined surgical procedures. The clinical and socio-demographic characteristics of the study population are presented in Table 1.

### 3.2. Short Form Health Survey (SF-12) Results

Table 2 presents the results of the SF-12 domains. Based on the mental component summary (MCS) score, in the present study, a priori risk of depression was identified in 29.03% of patients.

MCS score was not included in the subsequent analysis, due to its direct association with risk of depression. The results of the selection methods used are presented in Table 3.

### 3.3. Model of Risk of Depression

For the centroid class principal component analysis method, the best percentage of the total variance explained was 82.53%; thus, this model was used for further analyses. A model of feature selection was developed based on the centroids of the classes of the “depression risk” variable, which takes a value of 0 or 1.

**Figure 1:**
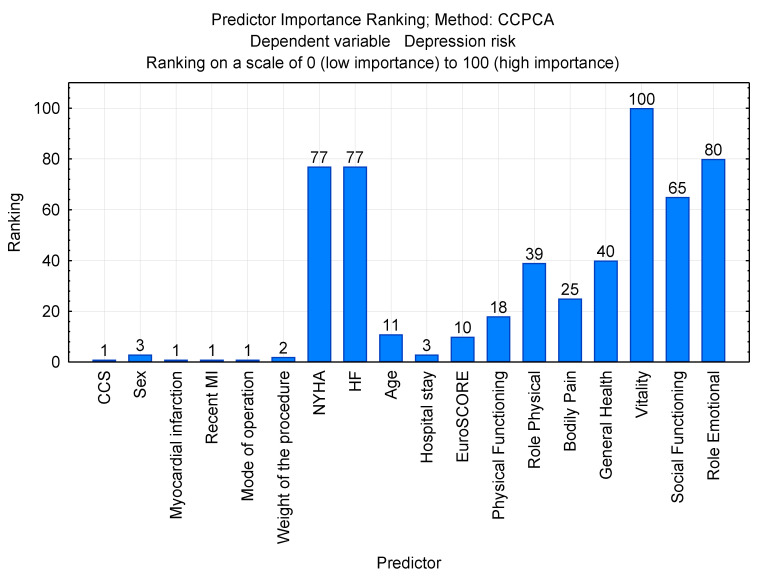
Results of features selection depending on discriminant power. CCS: Canadian Cardiovascular Society classification; NYHA: New York Heart Association classification of heart failure; HF: heart failure; EUROscore: European system for cardiac operative risk evaluation.

The ranking of the features obtained using the CCPCA method that discriminated the “depression risk” variable most strongly is presented in Figure 1.

The model indicated the highest importance for vitality, role-emotional, NYHA class, and heart failure (HF). Thus, these variables had the strongest influence on the risk of depression. The comparison of the correctness of the “depression risk” classification with the use of different classifiers depending on the number of principal components one to six is presented in Table 4. This approach allowed for the selection of the best final model.

**Figure 2:**
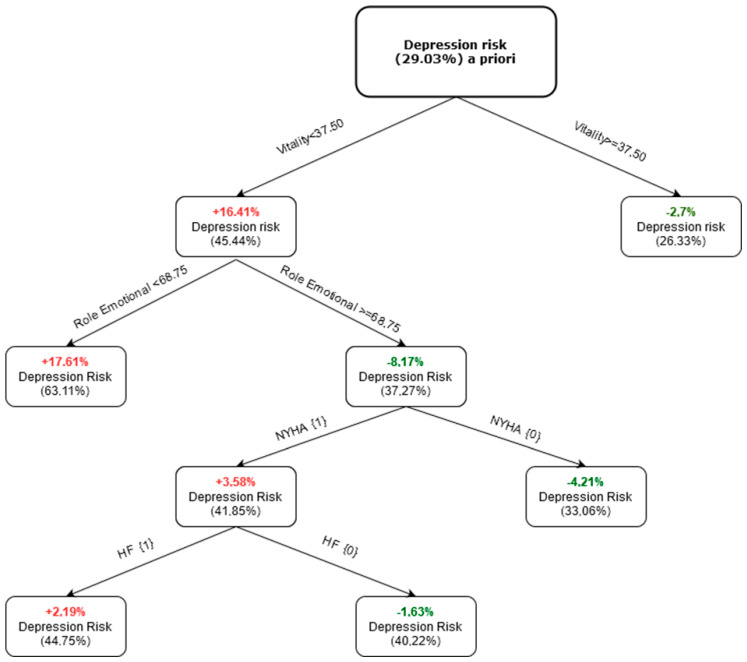
Classification and regression tree (CART). Depression risk a priori; risk of depression based on MCS score. NYHA; New York Heart Association classification of heart failure. HF; heart failure.

After using the various classifiers, we confirmed that the four identified principal components were the best solution for the “depression risk” assessment. The classification and regression trees type classification tree model, presented in Figure 2, was the best. With this strategy, four components allowed us to build the best model, in terms of discrimination against the above-mentioned features. The results of the eigenvalue and total explained variance are presented in Table 5.

On the basis of factor loadings λ > 0.7, we performed a final assignment of the measured parameters to each main component. These components are reflected in the CART model presented in Figure 2.

As shown in Figure 2, the risk of depression a priori in the studied sample was 29.03%. If the vitality score was ≥37.50, the risk of depression was the lowest, and the a posteriori risk of depression was 26.33%. The left arm of the CART tree significantly modified the risk of depression. If the vitality score was <37.50 and the role-emotional (RE) subscale score was <68.75 points, the risk of depression was the highest, at 63.11%. If the vitality score was <37.50 and role-emotional score was ≥68.75, the risk of depression was 37.27%. In this situation, the NYHA class and heart failure influenced the risk change. If NYHA was 0 (class I or II) and vitality was <37.50 and role-emotional score was >66.75, the risk of depression decreases by 4.21% to 33.06%. The risk of depression increased by 3.58% up to 41.85% when the NYHA class was one (class III or IV). In the latter situation, heart failure had an additional influence on the risk of depression. Despite the NYHA class (class III or IV), if a patient had no heart failure, the risk of depression decreased to 40.22%. In case of heart failure, the risk of depression increased up to 44.75%.

## 4. Discussion

Although objective clinical treatment outcomes are important, assessing patient-reported outcomes (PROs) is highly recommended to fully understand the determinants of well-being from the patient’s perspective [24]. Despite psychological factors being the most useful predictors and prognostic factors of quality of life in patients after cardiac surgery [25], in-depth studies are scarce. Moreover, the authors emphasized that focusing solely on objective parameters or functionality assessment cannot identify patients experiencing poor quality of life [24]. With a narrowed approach, subjective factors may be ignored, despite their proven importance.

Research on depression in cardiovascular diseases has indicated that it is a common and dangerous comorbidity [6,26]. The prevalence of depression illustrates the seriousness of the problem. However, knowing the risk factors seems of more importance, contributing to the identification of specific goals for therapeutic interventions [27]. Using machine learning methods, we constructed a model explaining the heightened risk of depression and indicating potential goals for therapy.

In the current study, an a priori risk of depression was observed in 29.03% of patients. A literature review indicated that the prevalence of depression after cardiac surgery is between 15% and 40%, and symptoms of depression can be identified in up to 60% [2,3,4,28,29]. Thus, our results are in line with those of other authors. Using machine learning allowed us to identify factors that are related to a significantly heightened risk of depression, increasing the possibility of a diagnosis of the disorder. The coexistence of low vitality and disturbed functioning due to emotional problems increased the risk of depression to 63.11%. An increase from an a priori risk of depression, however insignificant, was related to heart failure and its functional consequences, as measured with the NYHA classification. To the best of our knowledge, no study has yet used the same type of analysis among patients following cardiac surgery.

The results from longitudinal studies indicated more significant and prevalent improvements in physical health compared with mental health following cardiac surgery. Researchers, also using the SF-12 or SF-36, focused on dichotomous analyses, in which improvement or decline was noted [30]. The lack of more in-depth analyses has resulted in an insufficient understanding of the important factors determining treatment outcomes.

Auensen et al. [24] noted an improvement in the role-emotional domain in 36.9% of patients, compared with 61.5% of patients reporting an improvement in physical health, based on a minimal important difference analysis (MID). Ussia et al. [31] observed an improvement in physical functioning measured with the SF physical component summary (PCS), whereas the improvement in the mental component summary (MCS) after 3 months was not significant. Other authors emphasized that in a significantly large group of patients, no improvement with regard to the measured psychological parameters was observed [2,4,17].

We must pay more attention to the factors identified in our model, which may be undervalued or ignored in daily clinical practice. The factors strongly increasing the risk of depression were vitality, defined as energy and/or fatigue, and role-emotional, which reflects problems with daily activities resulting from emotional problems [18]. Special attention should be paid to a construct of fatigue that can be perceived as just a single symptom typical, especially in older patients with somatic illness [32]. Researchers have emphasized that fatigue is a multidimensional phenomenon that includes somatic, psychological, and existential dimensions, with highly subjective value and that can be an alarming symptom [33,34]. Our findings indicate that vitality and fatigue should be perceived as an important factor demanding more in-depth assessments. This suggestion is justified, as the vitality score is a predictor of treatment outcomes, including mortality [35]. Especially in patients with cardiovascular diseases, these features may be directly related to a somatic disease or may constitute a symptom of depression. An evident association between fatigue/vitality and decreased functioning related to emotional state is in line with this hypothesis. An evident association between depression and vitality was observed by Tully et al. [36]. Especially interesting aspects of exhaustion in patients with coronary heart disease as a vital concomitant symptom were emphasized by Appels [37]. Sustained fatigue, which becomes chronic in about 25% of patients, should be carefully examined because of its impact on difficulties with recovery and serious psychological suffering. Thus, fatigue should not be ignored; the assumption that it may be a “normal” symptom in patients after cardiac surgery, especially in older patients, is unjustified. The assessment of cognitive and emotional symptoms of depression can be highly effective. This can be achieved with the use of the hospital anxiety and depression scale (HADS); the visual analogue scale (VAS) can be successfully used to assess fatigue severity.

The identification of heart failure and NYHA functional class as important factors in our model indicates that physical functioning is also valuable. However, in the proposed model, psychological dimensions play a larger role. Identifying low vitality and its components, including emotional problems influencing daily functioning, would add substantial knowledge to allow more effective planning of therapy.

## 5. Study Strengths and Limitations

The current study has certain limitations. One of these is the overrepresentation of men in the studied sample. Future studies should be dedicated to women, as recommended in [27,38]. Another limitation is the lack of data on the previous treatment of mental disorders. It is possible that this fact may have importance. However, the aim of our study was to identify somatic factors and modifiable features related to depression risk, because they are more helpful for planning more effective interventions. We used the SF-12, a generic method for quality of life assessment. Using a disease-specific method was suggested as being more appropriate [17]. Although the concept of fatigue being one of the important symptoms in patients with depression is not new [34,39], our study showed its importance in another clinical context. The results of the current study justify screening for fatigue/low vitality and assessing its severity during routine assessment of patients following cardiac surgery.

The strengths of the current study lies in using machine learning, an advanced statistical analysis allowing more in-depth exploration of depression risk. This study adds to the literature through the construction of an especially effective depression risk model following cardiac surgery. An advantage also lies in the representative sample of patients following cardiac surgery procedures and using the standardized questionnaire recommended in studies involving patients with various somatic conditions, including cardiovascular. Recently, the need for further research on the physical and psychological nature of somatic/vegetative symptoms in patients after cardiac surgery was highlighted [40]. Our study, at least partially, fills this gap and justifies this postulate.

## 6. Conclusions

In the current study, we constructed a model of increased depression risk in patients following cardiac surgery, based on machine learning analysis. Assessing fatigue and vitality can help health professionals with identifying patients at risk of depression. In addition, assessing functional status and dimensions of fatigue, as well as the impact of emotions on daily functioning can help determine effective intervention options. Given our results, assessing fatigue and vitality is recommended during routine assessments of health status in patients following cardiac surgery.

## Figures and Tables

**Table 1:** Clinical and socio-demographic data of the studied population (*n* = 217).

	N (%) ^a^
Age; mean (SD)	65.14 (9.47)
Sex	
Women	75 (34.6)
Men	142 (65.4)
History of myocardial infarction	86 (39.6)
Hypertension	155 (71.4)
Diabetes mellitus	62 (28.6)
Recent myocardial infarction	50 (23.0)
History of percutaneous coronary intervention	54 (24.9)
Atrial fibrillation/flutter	27 (12.4)
Heart failure	59 (27.2)
Weight of the operation	
Isolated OPCABG ^b^/CABG ^c^	123 (56.7)
Single non-CABG	44 (20.3)
Combined 2 or 3 procedures	50 (23.0)
Extracorporeal circulation	97 (44.7)
Mode of operation	
Elective	133 (61.3)
Urgent	68 (31.3)
Emergent	16 (7.4)
Salvage	0 (0)
NYHA Class ^d^	
0–I	61 (28.1)
II–IV	156 (71.9)
CCS Class ^e^	
0–I	44 (20.3)
II–IV	173 (79.7)
EUROscore ^f^, mean (SD); median (min-max)	1.86 (1.31); 1.49 (0.5–8.26)
Hospital stay, mean (SD), median (min-max)	7.96 (2.68); 7 (5–25)

^a^ Data presented as number and percentage, unless otherwise specified; ^b^ OPCABG—off-pump coronary artery bypass grafting; ^c^ CABG—on-pump coronary artery bypass grafting; ^d^ NYHA—New York Heart Association classification of heart failure; ^e^ CCS—Canadian Cardiovascular Society classification; ^f^ EUROscore—European System for Cardiac Operative Risk Evaluation.

**Table 2:** Results of SF-12 domains and depression risk prevalence as derived from the MCS score.

SF-12 Domain	Mean (SD)
Physical Functioning (PF)	49.54 (30.80)
Role-Physical (RP)	48.67 (22.60)
Bodily Pain (BP)	60.48 (27.15)
General Health (GH)	47.86 (19.92)
Vitality (VT)	50.92 (25.78)
Social Functioning (SF)	64.19 (26.95)
Role-Emotional (RE)	65.44 (26.39)
Mental Health (MH)	64.98 (20.74)
PCS ^a^	40.94 (7.86)
MCS ^b^	47.87 (10.0)
Depression risk ^c^	63 (29.03)

^a^ PCS—SF-12 Physical Component Summary; ^b^ MCS—SF-12 Mental Component Summary; ^c^ Depression risk: <42 points in MCS.

**Table 3:** Results of selection with the use of five methods: percentage of total variance explained for the four principal components.

Method ^a^	% of Total Variance Explained
PCA	79.33
KPCA	80.66
GPCA	81.73
CCPCA	82.53
LDA	77.72

^a^ PCA; principal component analysis; KPCA; kernel principal component analysis; GPCA; gradient component analysis; CCPCA; centroid class principal component analysis; LDA; linear discriminant analysis.

**Table 4:** Statistics of the accuracy of the depression risk classification.

	NO	Centroid Class Principal Component AnalysisNumber of Principal Components					
		1	2	3	4	5	6
Regg ^a^	0.678	-	-	-	-	-	-
k-NN ^b^	0.713	0.717	0.711	0.721	0.712	0.718	0.711
SVM ^c^	0.711	0.723	0.761	0.767	0.732	0.723	0.702
MLP ^d^	0.732	0.739	0.745	0.782	0.803	0.766	0.743
CART ^e^	0.712	0.730	0.742	0.780	0.819	0.767	0.723
GNB ^f^	0.705	0.712	0.719	0.741	0.719	0.661	0.652

^a^ Regg: logistic regression; ^b^ k-NN: k-nearest neighbor; ^c^ SVM: support vector machine; ^d^ MLP: multilayer perceptron; ^e^ CART: classification and regression tree; ^f^ GNB; naive Bayes classifier.

**Table 5:** Basic statistics for each of the four principal components selected using the CCPCA ^a^ method.

Component	Eigenvalue	% of Total Variance	Cumulative Eigenvalue	Cumulative %
1	3.73	31.55	3.73	31.55
2	2.33	27.55	6.06	59.10
3	2.19	15.33	8.25	74.43
4	1.44	7.43	9.69	81.86

^a^ CCPCA: centroid class principal component analysis.

## Data Availability

Data supporting the results are available by contacting the corresponding author.
